# Evolution of tricuspid valve regurgitation after implantation of a leadless pacemaker: A single center experience, systematic review, and meta‐analysis

**DOI:** 10.1111/jce.15565

**Published:** 2022-06-07

**Authors:** Andreas Haeberlin, Joanna Bartkowiak, Nicolas Brugger, Hildegard Tanner, Elaine Wan, Samuel H. Baldinger, Jens Seiler, Antonio Madaffari, Gregor Thalmann, Helge Servatius, Laurent Roten, Fabian Noti, Tobias Reichlin

**Affiliations:** ^1^ Department of Cardiology, Inselspital, Bern University Hospital University of Bern Bern Switzerland; ^2^ Sitem Center for Translational Medicine and Biomedical Entrepreneurship University of Bern Bern Switzerland; ^3^ Division of Cardiology, Department of Medicine, Vagelos College of Physicians and Surgeons Columbia University New York New York USA

**Keywords:** atrio‐ventricular, leadless pacemaker, Micra, tricuspid regurgitation, tricuspid valve

## Abstract

**Introduction:**

Conventional transvenous pacemaker leads may interfere with the tricuspid valve leaflets, tendinous chords, and papillary muscles, resulting in significant tricuspid valve regurgitation (TR). Leadless pacemakers (LLPMs) theoretically cause less mechanical interference with the tricuspid valve apparatus. However, data on TR after LLPM implantation are sparse and conflicting. Our goal was to investigate the prevalence of significant TR before and after LLPM implantation.

**Methods:**

Patients who received a leadless LLPM (Micra™ TPS, Medtronic) between May 2016 and May 2021 at our center were included in this observational study if they had at least a pre‐ and postinterventional echocardiogram (TTE). The evolution of TR severity was assessed. Following a systematic literature review on TR evolution after implantation of a LLPM, data were pooled in a random‐effects meta‐analysis.

**Results:**

We included 69 patients (median age 78 years [interquartile range (IQR) 72–84 years], 26% women). Follow‐up duration between baseline and follow‐up TTE was 11.4 months (IQR 3.5–20.1 months). At follow‐up, overall TR severity was not different compared to baseline (*p* = .49). Six patients (9%) had new significant TR during follow‐up after LLPM implantation, whereas TR severity improved in seven patients (10%). In the systematic review, we identified seven additional articles that investigated the prevalence of significant TR after LLPM implantation. The meta‐analysis based on 297 patients failed to show a difference in significant TR before and after LLPM implantation (risk ratio 1.22, 95% confidence interval 0.97–1.53, *p* = .11).

**Conclusion:**

To date, there is no substantial evidence for a significant change in TR after implantation of a LLPM.

## 1 INTRODUCTION

Tricuspid valve regurgitation (TR) aggravates heart failure,^1^, ^2^ and is associated with impaired prognosis independent of a patient's age, left ventricular function or the presence of pulmonary hypertension.^3^, ^4^ After implantation of a conventional pacemaker, the likelihood of significant TR (defined as at least moderate TR) is more than twice as high as compared with patients without device.^5^ Besides a pacing‐induced lead‐independent functional increase of TR, TR aggravation seems to be caused mainly by ventricular leads of conventional cardiac pacemakers. Transvenous leads can interfere mechanically with tricuspid valve leaflets, tendinous chords and papillary muscles.^6^ This may lead to impaired mobility and insufficient leaflet coaptation, resulting in significant TR.

Leadless cardiac pacemakers (LLPMs) have been introduced to prevent lead‐associated complications of conventional pacemakers.^7^ Direct mechanical interference of LLPMs with the tricuspid valve seems less likely due to the limited size of the devices and the lack of structures permanently crossing the tricuspid valve. However, data on the evolution of TR after LLPM implantation remain sparse and conflicting. Some authors have reported a reduction or a stable prevalence of significant TR after LLPM implantation,^8^, ^9^, ^10^ while others observed a clear increase of significant TR afterwards.^11^, ^12^

In this study, we analyzed the degree and predictors for changes in TR severity after LLPM implantation at our center. Subsequently, a systematic literature review was performed and our data were pooled to perform a meta‐analysis on the prevalence of significant TR after LLPM implantation.

## 2 METHODS

### 2.1 Study design and patient population

In this investigator‐initiated observational study, we analyzed prospectively collected data from all patients that had received a LLPM (Micra™ TPS, Medtronic) at our tertiary referral center between May 2016 and May 2021. All patients had a guideline‐conformant indication for a pacemaker. The decision to implant a leadless system instead of a conventional device was made individually based on the patient's co‐morbidity and preference. The study was approved by the local ethics committee and conducted according to the principles of the Declaration of Helsinki.

### 2.2 Implantation procedure and follow‐up data acquisition

The LLPM implantation was performed according to standard practice.^13^ All interventions were performed by trained electrophysiologists, who had undergone the implantation training recommended by the manufacturer. The implantations were performed under fluoroscopic guidance, no peri‐interventional echocardiography was performed. Right and left anterior oblique fluoroscopic projections were used to identify a suitable position for LLPM deployment.

Detailed data of the implantation procedure were acquired prospectively (e.g., patient history, pacemaker indication, pre‐interventional transthoracic echocardiography (TTE), and procedural implant and device data). Follow‐up data were collected from the hospitals' electronic records and from external cardiologists. TTE studies were performed according to established guidelines,^14^ tricuspid valve regurgitation (TR) was evaluated using an integrative multiparametric approach and was graded as 0 = none, 1 = trace/mild, 2 = moderate and 3 = severe. TR of grade >1 was defined to be significant.^15^

### 2.3 Systematic review and study inclusion criteria

A systematic literature review was conducted in December 2021 in PubMed and Embase. The following Boolean search terms were used: “leadless,” or “Micra” or “Nanostim” and “tricuspid.” Subsequently, titles and abstracts were reviewed. Case reports, editorials, reviews, and letters were excluded. Full text reviews of nonexcluded articles were performed subsequently. Articles were included if they reported TR severity at least once pre‐ and postinterventionally after LLPM implantation. Studies that were performed ex vivo, in animals, or that were associated with simultaneous repair/replacement of the tricuspid valve were excluded as well. Whenever multiple articles were published from the same underlying cohort (e.g., publication of preliminary congress abstracts), we removed this double hits and only included the article with the largest patient population. The reference list of eligible articles was cross‐checked for additional literature that was not identified previously.

We aimed to analyze the prevalence of significant TR in a meta‐analysis. TR of moderate and severe degree as reported in the included studies was considered significant. Whenever the “moderate” category was further subdivided into “moderate to severe” or “mild to moderate,” we also considered these categories as significant TR.

### 2.4 Statistical analysis

Categorical variables are expressed as numbers and percentages. Continuous variables are shown as median and interquartile range (IQR). Comparisons of echocardiography data before and after LLPM implantation were performed using Wilcoxon's signed‐rank test or—in the case of paired categorical data—using the Stuart–Maxwell test for marginal homogeneity.

To identify predictors for TR increase, uni‐ and multivariate logistic regression models were fitted. The multivariate model included all variables from the univariate models with a *p*‐value < .1.

For the meta‐analysis, summary estimates were calculated by pooling the individual estimates of all included studies using inverse‐variance weights obtained from a random‐effects meta‐analysis. The random‐effect method was chosen due to several factors. First, the data were gathered from published literature and our single‐center analysis. Second, because implantation strategy may have changed over time and study populations differ. Finally, our goal was to provide a generalizable estimate of the treatment effect. Separate meta‐analyses were performed for both available LLPM systems (i.e., Micra™ TPS and Nanostim™; Abbott Medical Inc.).

R version 4.1.1 for Windows (R Foundation) including the package “meta” (for the meta‐analysis) and SPSS version 25 (IBM) were used. A *p* value< .05 was considered significant.

## 3 RESULTS

### 3.1 Baseline demographic and procedural details

Of all 116 patients that underwent LLPM implantation in the respective timeframe, 69 (59%) had at least one TTE exam before and after LLPM, which allowed grading of TR. The baseline characteristics of the 69 included patients and the respective procedural data are shown in Table 1. Two patients (3%) experienced complications during LLPM implantation that required further interventions: one patient developed cardiac tamponade several hours after the intervention, which was performed under oral anticoagulation. The patient required pericardiocentesis, the drainage was removed the following day and the patient was released from hospital 3 days later. Another patient had a bleeding from the femoral vascular insertion site (pseudoaneursym). Sonography‐controlled local thrombin injection was performed.

**Table 1:** Baseline characteristics

Patient and procedural characteristics	*n* = 69
**Clinical patient characteristics and comorbidities**	
‐Age [years]	78 (72–84)
‐Female gender [*n*]	18 (26%)
‐Body height [m]	1.70 (1.64–1.75)
‐Body mass index [kg/m^2^]	26.1 (22.9–29.7)
‐NYHA class	2 (1–3)
‐Coronary artery disease [*n*]	32 (46%)
‐Arterial hypertension [*n*]	56 (81%)
‐Diabetes [*n*]	20 (29%)
‐Dyslipidemia [*n*]	33 (48%)
‐Chronic kidney disease (GFR < 60 ml/min) [*n*]	38 (55%)
‐Aortic valve replacement before LLPM implantation [*n*]	25 (36%)
o Patients with aortic valve replacement before baseline echo	11 (16%)
o Patients with aortic valve replacement after baseline echo	14 (20%)
‐Mitral valve repair before LLPM implantation [*n*]	1 (1%)
o Patients with mitral valve repair before baseline echo	1 (1%)
o Patients with aortic valve repair after baseline echo	0 (0%)
Medication	
‐Betablockers [*n*]	39 (57%)
‐Class III antiarrhythmic drugs [*n*]	7 (10%)
‐Antiplatelet therapy [*n*]	28 (41%)
‐Oral anticoagulants [*n*]	47 (68%)
‐Antihypertensive drugs [*n*]	50 (72%)
Pacemaker indication	
‐Atrial tachyarrhythmia and planned AV node ablation [*n*]	15 (22%)
‐Permanent 3^rd^ degree AVB [*n*]	13 (19%)
‐Intermittent 3^rd^ degree AVB [*n*]	15 (22%)
‐Intermittent high‐degree AVB [*n*]	4 (6%)
‐Symptomatic second‐degree AVB [*n*]	1 (1%)
‐Left bundle branch block + 1^st^ degree AVB [*n*]	3 (4%)
‐Sick sinus syndrome [*n*]	2 (3%)
‐Atrial fibrillation associated bradycardia [*n*]	10 (14%)
‐Other [*n*]	6 (9%)
Procedure duration and fluoroscopy time/dosage	
‐Procedure duration [min]	51 (42–68)
‐Fluoroscopy duration [min]	6.2 (4.6–9.8)
‐Radiation dose [cGycm^2^]	1'478 (800–3'312)
Implantation characteristics	
‐Number of engaged tines [*n*]	2 (2–3)
‐Number of required pacemaker deployments [*n*]	1 (1–2)
o 1 deployment [*n*]	39 (57%)
o 2 deployments [*n*]	15 (22%)
o >2 deployments [*n*]	13 (19%)
‐Final implantation site	50 (72%)
o Septum [*n*]	15 (22%)
o Apex [*n*]	3 (4%)
o RVOT [*n*]	1 (1%)
o Free wall [*n*]‐Used volume of contrast medium [ml]	25 (15–40)
Acute electrical implantation characteristics	
‐Pacing threshold [V/0.24 ms]	0.38 (0.38–0.63)
‐Sensed R‐wave amplitude [mV]	9.9 (7.5–13.72)
‐Pacing impedance [Ω]	730 (640–850)

*Note*: Median values with interquartile ranges in brackets and numbers with percentages are shown.

Abbreviations: AVB, AV block; GFR, glomerular filtration rate; LVEF, left ventricular ejection fraction; LVEDD, left ventricular end‐diastolic diameter; LVMI, left ventricular mass index; NYHA, New York Heart Association; RVOT, right ventricular outflow tract; TAPSE, tricuspid annular plane systolic excursion.

### 3.2 Echocardiographic assessment

**Figure 1:**
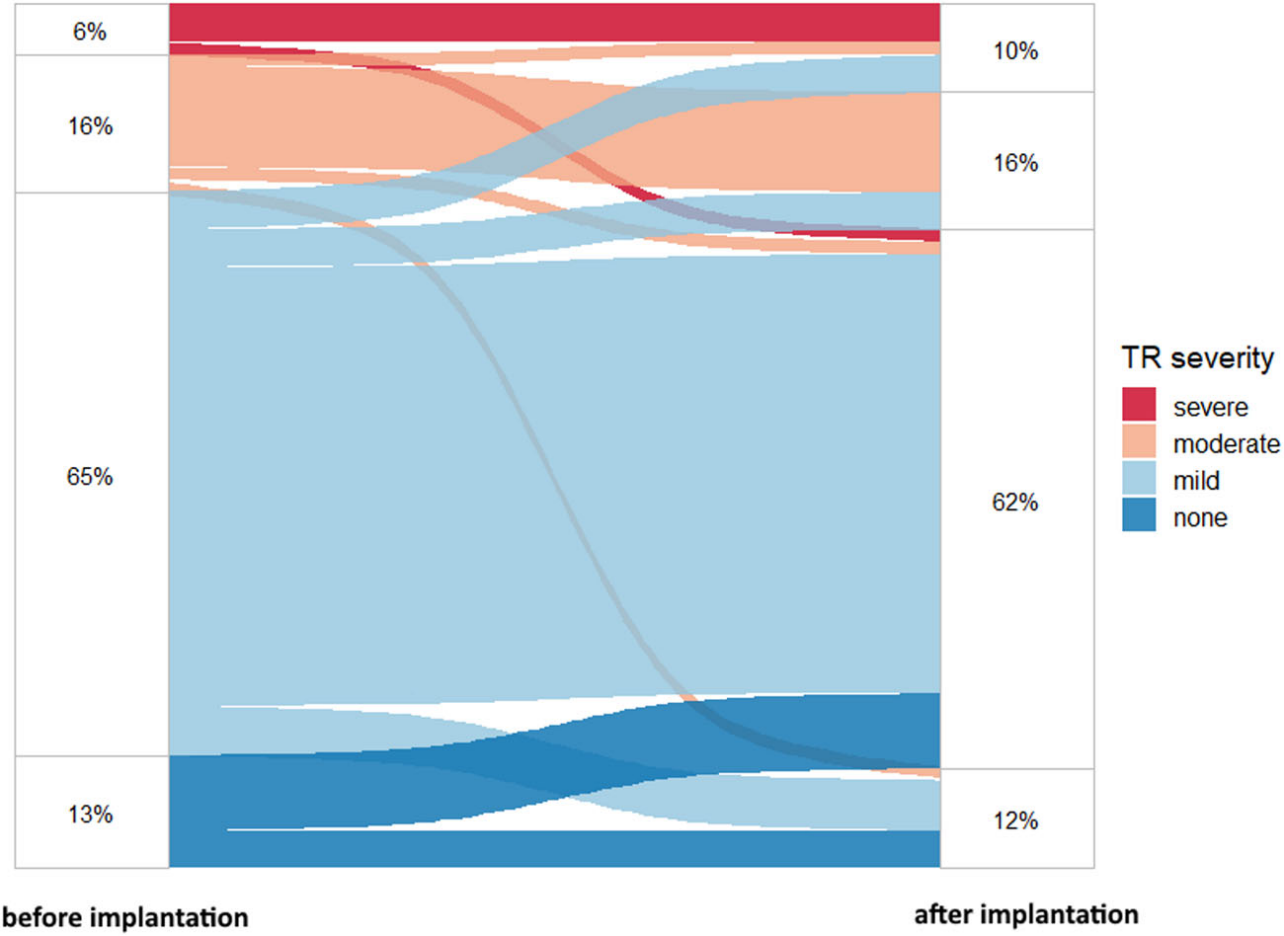
Alluvial diagram of tricuspid valve regurgitation (TR) severity before and after leadless pacemaker (LLPM) implantation. At baseline, nine patients had no TR, 45 patients had mild TR, 11 patients had moderate TR, and four patients had severe TR. During follow‐up, eight patients had no TR, 43 patients had mild TR, 11 patients had moderate TR, and seven patients had severe TR.

Detailed echocardiography data collected at baseline and during follow‐up are shown in Table 2. Follow‐up duration between baseline and follow‐up TTE was 11.4 months (interquartile range [IQR] 3.5–20.1 months). Over this time, the mean aortic valve pressure gradient assessed by Doppler echocardiography changed significantly, related to aortic valve implantation, which was performed in 25 (36%) of patients. There was no change in TR severity (*p* = .49). Detailed trends of TR severity before and after LLPM implantation are summarized in Figure 1. Significant TR was present in 15 patients (22%) before and 18 patients (26%) after LLPM implantation. TR worsened at follow‐up in 13 patients (19%). Six of these patients (9%) evolved from none to mild TR, and one patient (1%) with previously moderate TR developed severe TR. Three patients (4%) with mild TR developed moderate TR and another three patients (4%) developed severe TR. Thus, six patients (9%) had new significant TR during follow‐up after LLPM implantation. In contrast, TR severity improved in seven patients (10%), of which two (29%) had also received aortic valve replacement.

**Table 2:** Echocardiography data before and after LLPM implantation

Echocardiography data	Before implantation	During follow‐up	*p* value
Left ventricle, right ventricle, and left atrium			
– LVEF [%]	60 (55–60)	60 (50–65)	.960
– LVEDD [mm]	46 (42–51)	47 (43–50)	.903
– Interventricular septum thickness [mm]	13 (11–14)	12 (11–14)	.237
– LVMI [g/m^2^]	116 (86–136)	116 (93–142)	.926
– Tricuspid annulus diameter [mm]	37 (32–42)	39 (35–45)	.423
– TAPSE [mm]	17 (14–19)	16 (14–20)	.793
– FAC [%]	41 (34–54)	35 (32–42)	.250
– LAVI [ml/m^2^]	51 (38–69)	54 (43–68)	.194
– RV/RA gradient [mmHg]	32 (25–44)	32 (25–38)	.117
Valve function			
o Tricuspid valve regurgitation			.49
o None [*n*]	9 (13%)	8 (12%)	
o Mild [*n*]	45 (65%)	43 (62%)	
o Moderate [*n*]	11 (16%)	11 (16%)	
o Severe [*n*]	4 (6%)	7 (10%)	1
Mitral valve regurgitation			
None [*n*]	6 (9%)	6 (9%)	
Mild [*n*]	53 (77%)	52 (75%)	
o Moderate [*n*]	10 (14%)	11 (16%)	
o Severe [*n*]	0 (0%)	0 (0%)	
o Aortic valve regurgitation			.56
o None [*n*]	27 (49%)	31 (51%)	
o Mild [*n*]	27 (49%)	28 (46%)	
o Moderate [*n*]	0 (0%)	2 (3%)	
o Severe [*n*]	1 (2%)	0 (0%)	
Aortic valve stenosis			.081
o None [*n*]	37 (63%)	48 (87%)	
o Mild [*n*]	7 (12%)	1 (2%)	
o Moderate [*n*]	7 (12%)	5 (9%)	
o Severe [*n*]	8 (14%)	1 (2%)	
o Mean gradient [mmHg]	13 (5–21)	9 (6–12)	**.011**

*Note*: Median values with interquartile ranges in brackets and numbers with percentages are shown.

Abbreviations: FAC, fractional area change; LA, left atrial; LAVI, left atrial volume index; LVEF, left ventricular ejection fraction; LVEDD, left ventricular end‐diastolic diameter; LVMI, left ventricular mass index; NYHA, New York Heart Association; RA, right atrium; RV, right ventricle; TAPSE, tricuspid annular plane systolic excursion.

Patients with >20% ventricular pacing at follow‐up (51 patients, 74%) more often had a higher degree of TR than patients that were rarely paced (*p* = .009). Moderate TR was present in 20%, severe TR in 14% of patients with >20% ventricular pacing, whereas in patients who were rarely paced, only one patient (6%) had moderate TR and none suffered from severe TR.

### 3.3 Predictors for increase of tricuspid valve regurgitation in the presence of a LLPM

Predictors for an increase of TR are shown in Table 3. Univariate predictors for TR worsening include a higher baseline RV/RA gradient (*p* = .02) and a higher percentage of ventricular pacing during follow‐up (*p* = .06). Prior valve replacement surgery (before LLPM implantation) reduced the risk for TR worsening (*p* = .06). In the multivariate analysis, a higher RV/RA gradient remained predictive for worsening of TR severity.

**Table 3:** Predictors for increase of tricuspid valve regurgitation

	Univariate analysis		Multivariate analysis	
Variables	OR (95% CI)	*p* value	OR (95% CI)	*p* value
Patient‐related factors				
‐Age	0.96 (0.90–1.03)	.30	–	–
‐Male gender	2.20 (0.44–11.05)	.34	–	–
‐BMI	1.03 (0.94–1.15)	.50	–	–
‐NYHA class	1.20 (0.59–2.45)	.61	–	–
‐Coronary artery disease	0.99 (0.29–3.32)	.99	–	–
‐Prior myocardial infarction	0.78 (0.03–2.32)	.24	–	–
‐Prior valve replacement	0.26 (0.06–1.05)	**.06**	2.16 (0.23–20.38)	.50
‐Arterial hypertension	0.72 (0.17–3.12)	.67	–	–
‐Diabetes	2.57 (0.74–8.95)	.14	–	–
‐Dyslipidemia	1.35 (0.40–4.52)	.63	–	–
‐Chronic kidney disease	2.30 (0.26–19.95)	.45	–	–
‐LVEF	0.98 (0.93–1.04)	.50	–	–
‐LVEDD	1.04 (0.95–1.13)	.40	–	–
‐LVMI	1.01 (0.99–1.02)	.43	–	–
‐Interventricular septum thickness	1.17 (0.87–1.58)	.30	–	–
‐LAVI	1.01 (0.99–1.04)	.26	–	–
‐RV/RA gradient	1.08 (1.01–1.15)	**.02**	1.09 (1.01–1.18)	**.02**
‐Tricuspid annulus diameter	1.04 (0.92–1.17)	.58	–	–
‐TAPSE	0.99 (0.87–1.12)	.85	–	–
‐FAC	1.02 (0.94–1.10)	.68	–	–
Medication				
‐Betablockers	1.29 (0.38–4.44)	.69	–	–
‐Class III antiarrhythmic drugs	1.85 (0.32–10.83)	.49	–	–
‐Antiplatelet therapy	1.94 (0.58–6.57)	.28	–	–
‐Oral anticoagulants	1.07 (0.29–3.93)	.92	–	–
‐Antihypertensive drugs	5.68 (0.69–47.15)	.11	–	–
Pacemaker indication				
‐Atrial tachyarrhythmia and planned AV node ablation	0.60 (0.12–3.07)	.54	–	–
‐Permanent 3rd degree AVB	1.38 (0.32–5.94)	.66	–	–
‐Intermittent 3rd degree AVB	1.82 (0.47–7.01)	.39	–	–
‐Left bundle branch block + 1st degree AVB	2.25 (0.19–26.89)	.52	–	–
‐Sick sinus syndrome	4.58 (0.27–78.55)	.29	–	–
‐Atrial fibrillation associated bradycardia	1.09 (0.20–5.87)	.92	–	–
Procedure‐related factors				
‐Procedure duration	1.00 (0.97–1.03)	.97	–	–
‐Number of PM deployments	0.96 (0.62–1.50)	.87	–	–
‐Non‐septal implantation site	0.75 (0.18–3.09)	.69	–	–
‐Number of engaged LLPM tines	1.71 (0.69–4.26)	.25	–	–
PM‐related factors				
‐Percentage of ventricular pacing during follow‐up	1.02 (1.0–1.05)	**.06**	1.07 (0.99–1.15)	.11

*Note*: Prior valve replacement refers to valve interventions that were performed before LLPM implantation.

Abbreviations: AVB, AV block; CI, confidence interval; LVEF, left ventricular ejection fraction; LVEDD, left ventricular enddiastolic diameter; OR, odds ratio; LLPM, leadless pacemaker; NYHA, New York Heart Association; TAPSE, tricuspid annular plane systolic excursion.

### 3.4 Systematic literature review

**Figure 2:**
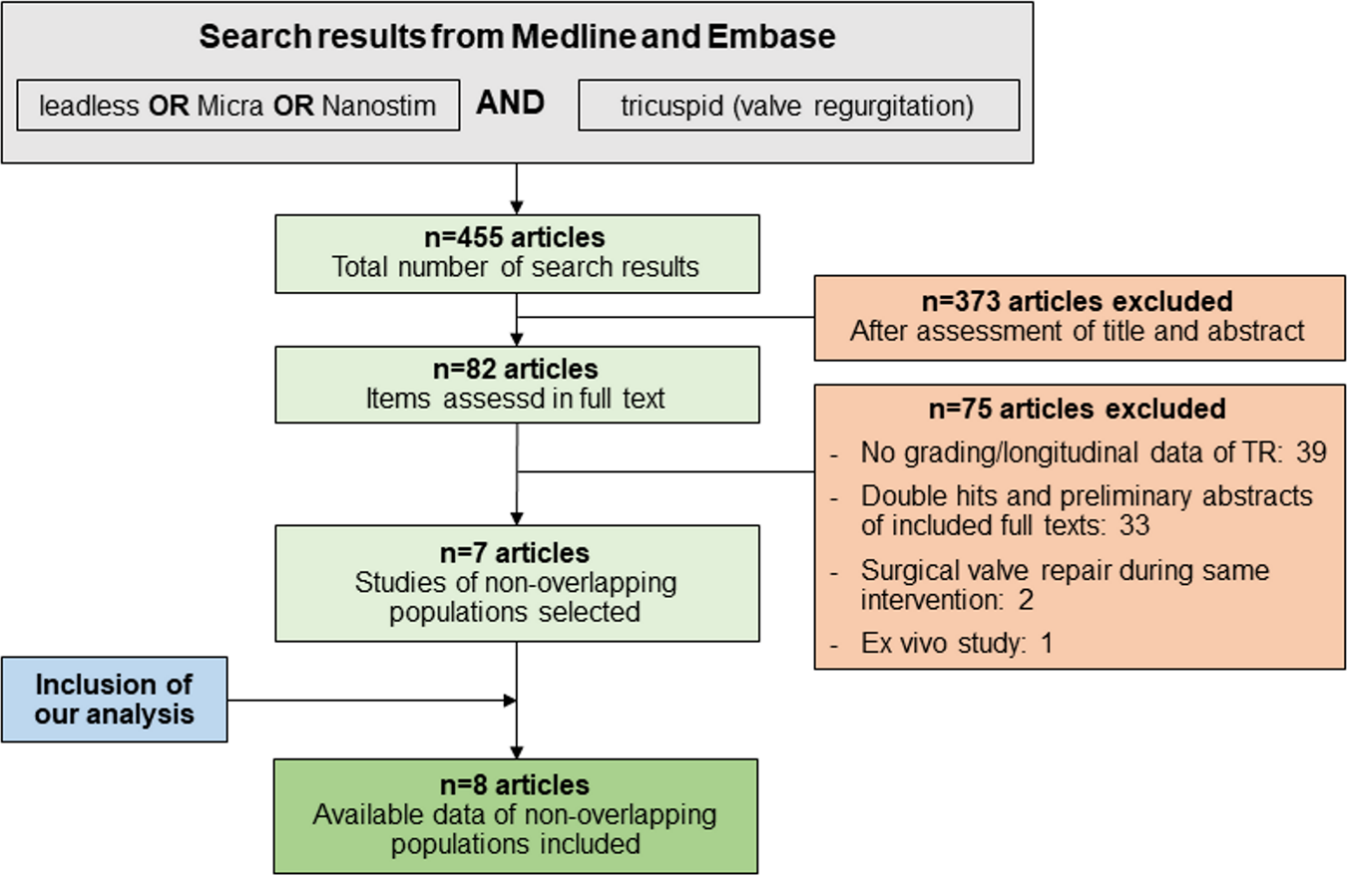
Study identification and selection procedure.

Based on the search terms, we identified 455 articles that underwent screening on title and abstract level. Eighty‐two studies were further examined in full text, and seven met the pre‐specified inclusion criteria (Figure 2).^8^, ^9^, ^11^, ^12^, ^16^, ^17^, ^18^ The included studies from 2016 to 2022 were mostly of retrospective nature and encompassed data from 297 patients: 255 patients with a Micra™ LLPM and 42 patients with a Nanostim™ LLPM, whereof 42% (Micra™) and 32% (Nanostim™) were women. Mean age was 79.8 ± 2.5 years for Micra™ and 81.0 ± 1.4 years for Nanostim™ patients. The patients had preserved left ventricular ejection fraction (LVEF 57.3% ± 3.8% [Micra™]/54.9% ± 2.3% [Nanostim™]) and a mean follow‐up duration was 12.7 ± 3.9 months (Micra™) and 11 ± 3 months (Nanostim™). Study details, including risk factors for TR worsening and the proportion of patients that showed change in TR, are summarized in Table 4.

**Table 4:** Description of the studies included in the meta‐analysis

Study	Design	Study size	Mean age [y]	Female [%]	LVEF pre implant [%]	Vent. pacing @FU [%]	Septal implant site [%]	% of patients with TR worsening	% of patients with TR improvement	Risk factors for worsening of TR	Mean FU duration [months]
Garikipati et al.^16^	n.a.	22 Micra™	77 ± 9	50%	56 ± 9	n.a.	n.a.	n.a.	n.a.	n.a.	n.a.
Salaun et al.^17^	P	9 Micra™	85 ± 6	52%	62 ± 13	34 ± 42	74%	4%	4%	Pulmonary artery pressure?	2
		14 Nanostim™									
Beurskens et al.^11^	R	25 Micra™	81 ± 8	30%	53 ± 10	~46*	21%*	43%	6%	Septal implantation (*p* = .03), distance of LLPM to tricuspid valve (*p* = .09)	12
		28 Nanostim™									
					54 ± 9						
Moore et al.^8^	R	10 Micra™	83 ± 10*	40%	53	43 ± 41*	50%	20%	30%	n.a.	5.2 ± 1.9
Theis et al.^18^	R	14 Micra™	78 ± 4	29%	52 ± 6	88 ± 12	93%	0%	n.a.	None	n.a.
Dabas et al.^9^	R	42 Micra™	76 ± 4	55%	n.a.	n.a.	n.a.	8%	21%	n.a.	n.a.
Hai et al.^12^	P	64 Micra™	81 ± 9	52%	61 ± 8	n.a.	100%	19%	n.a.	Distance between LLPM and tricuspid annulus (*p* = .03)	15
Haeberlin et al.^13^	R	69 Micra™	78 ± 9	26%	56 ± 10	67 ± 40	72%	19%	10%	RV/RA gradient (*p* = .02), ventricular pacing (*p* = .11)	15 ± 14
Micra™		255 Micra™	80 ± 3	42%	57 ± 4	53 ± 12	70 ± 30				12.7 ± 3.9
Nanostim™											
		42 Nanostim™		32%							
					55 ± 2	45 ± 4	23 ± 16				11 ± 3.0
			81 ± 1								
**Overall analysis**			**80** ± **2**	**40%**	**56** ± **4**	**49** ± **11**	**58** ± **33**	**25%**	**11%**		**12.1** ± **3.9**

*Note*: Asterisks indicate data, which were not directly reported in the respective manuscripts but were derived from additionally provided data/data supplements. The overall summaries provide a weighted mean (derived from the weights of the pooled random‐effect meta‐analyses).

Abbreviations: FU, follow‐up; LVEF, left ventricular ejection fraction; n.a., not available.

### 3.5 Meta‐analysis of occurrence of significant TR after LLPM implantation

**Figure 3:**
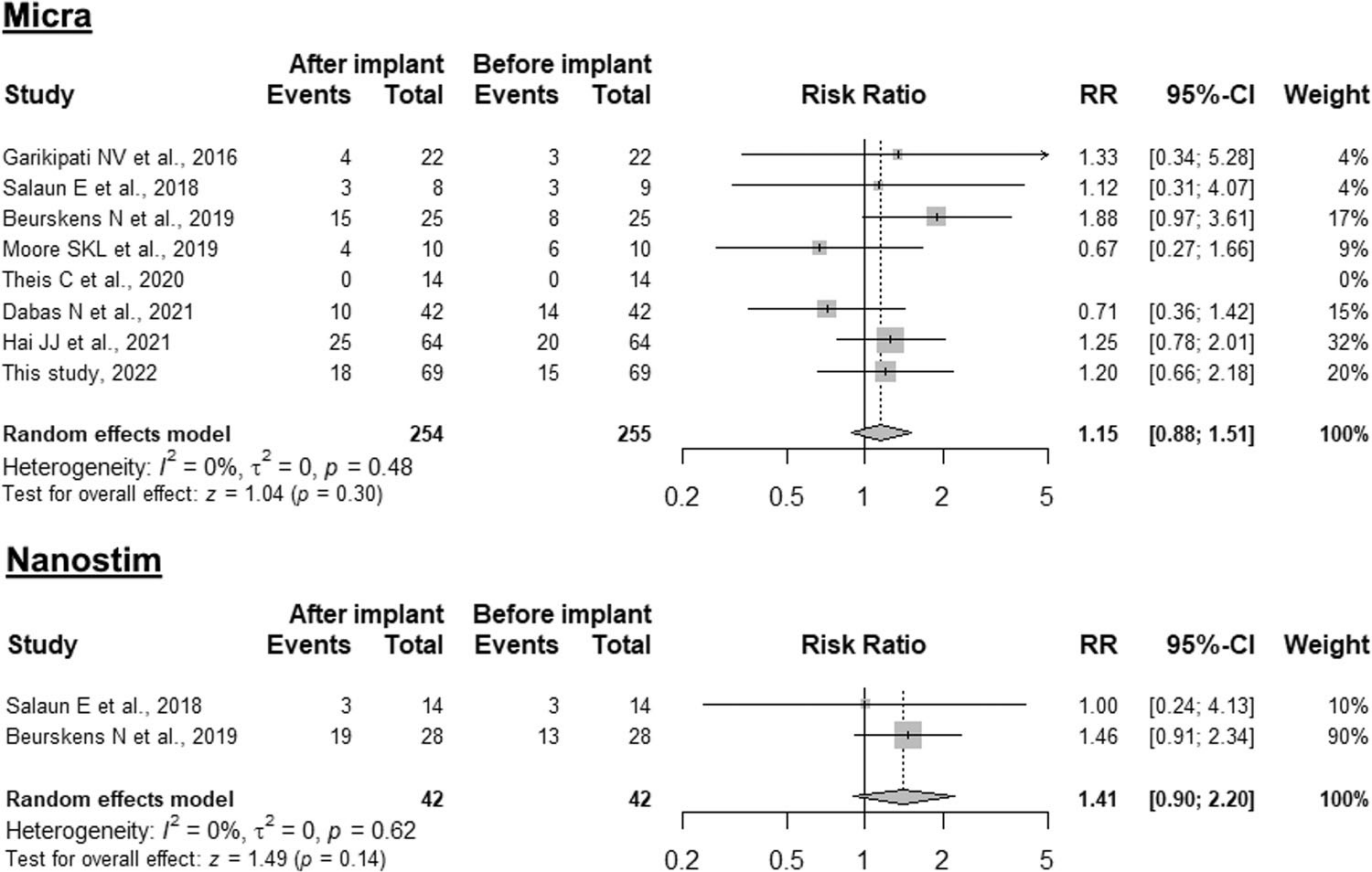
Meta‐analysis of tricuspid valve regurgitation of moderate or severe degree (labeled as “event”) before and after implantation of a LLPM (top panel: Micra™ TPS; bottom panel: Nanostim™). Horizontal lines represent 95% confidence intervals of the estimate. Studies are ordered from top to bottom according to the year of publication. CI, confidence interval; RR, risk ratio.

Figure 3 shows the prevalence of a significant TR (Grade ≥ 2) before and after LLPM implantation across all included studies. The overall mean prevalence of significant TR was 27.1% before pacemaker implantation. After LLPM implantation and a median follow‐up duration of 12.1 months, significant TR was found in 31.1% of patients. Separate meta‐analyses are provided for both commercially available LLPM systems. No statistical differences in the prevalence of significant TR before and after LLPM implantation was observed for Micra™ (risk ratio 1.15, 95% confidence interval [CI] 0.88–1.51, *p* = .3) and Nanostim™ (risk ratio 1.41, 95% CI 0.90–2.20, *p* = .14) devices. Similarly, in the overall pooled estimate including data from all LLPM patients (Micra™ and Nanostim™; *n* = 297), the prevalence of significant TR before and after LLPM implantation was not different (risk ratio 1.22, 95% CI 0.97–1.53, *p* = .11). No significant heterogeneity was present (*I* ^2^ = 3%, *p* = .48). According to the corresponding funnel plot (supplementary figure), there was no evidence of publication bias.

## 4 DISCUSSION

In this article, we assessed the evolution of TR severity after implantation of a LLPM. Besides our single‐center data, we provide the first systematic review and meta‐analysis on the prevalence of significant TR after LLPM implantation. The main findings of this study are:

(1) The prevalence of significant TR before and after LLPM implantation is relatively high (~30%), likely attributable to the elderly and multimorbid patient population that undergoes LLPM implantation.
(2) Changes in severity of TR after LLPM implantation are not uncommon but the majority of patients show unchanged (71%) or improved (10%) tricuspid valve function.
(3) Based on the meta‐analysis of available data, there is no evidence for an increase in the prevalence of significant TR up to one year after LLPM implantation.

### 4.1 Mechanisms of interference between LLPMs and the tricuspid valve

Acute implantation‐procedure‐related damage of the tricuspid valve might be caused during the maneuvering of the device into the right ventricular (RV) cavity and/or during repositioning of the LLPM. However, there are no data assessing the incidence of such events. In an ex vivo study, Mattson et al. demonstrated that damage of the subvalvular apparatus caused by the Micra™ fixation tines in the case of device repositioning is unlikely to occur.^19^ Consistently, the number of required LLPM deployments did not predict increase of TR in our study, which was also not observed by others.^11^, ^12^ In contrast, it has been hypothesized that a septal instead of an apical implantation site may be a mechanical contributor to tricuspid valve dysfunction. Based on the findings of Hai et al.^12^ and our own data, a septal implantation did not lead to TR increase. The increased risk of TR worsening after septal LLPM implantation that was reported by Beurskens et al. ^11^ may be driven by the high number of Nanostim™ implants in this study, which are significantly longer and may interfere with the valve.^17^ Numerical models have emphasized the significant influence of large LLPM housings on the collision likelihood with adjacent cardiac structures.^20^ Thus, while a septal implantation of a Micra™ alone may not be risk factor for TR worsening, a very basal implantation site close to the tricuspid valve annulus should be avoided to preserve tricuspid valve function.^12^

Besides direct negative mechanical interference of LLPMs with the tricuspid valve, ventricular pacing may per se induce valve dysfunction due to the nonphysiologic electrical activation pattern, which has been shown in conventional pacemaker patients undergoing RV apical pacing.^21^ Data regarding this functional impact on TR are conflicting, but the influence of ventricular stimulation is inevitable if patients require stimulation.

### 4.2 Impact on TR of LLPMs versus conventional transvenous pacemakers

Conventional transvenous pacemakers may cause worsening of TR already early after device implantation.^22^ While the overall risk of TR worsening is limited, the prevalence of significant TR may be increased by a factor of up to 2.3 following transvenous device implantation.^5^ This is substantially more than the (nonsignificant) risk ratio we identified in the meta‐analyses on LLPMs.

There are no randomized data available on the effect on TR depending on the implantation of a LLPM or a conventional pacemaker. However, studies have investigated the impact on TR of both approaches by matching LLPM patients to a conventionally implanted cohort. Vaidya et al. compared 90 LLPM patients (Micra™ and Nanostim™ implants) 1:1 to age and sex‐matched control group.^23^ They observed a higher increase in TR severity in patients who had received conventional pacemakers than patients with LLPMs. The median follow‐up duration of this study was just 2 months, allowing only to draw limited conclusions on acute effects of the devices on valve function. In a smaller study of 53 patients, who were also matched for age and sex, Beurskens et al. described worsening of TR in 43% with LLPM versus 38% patients with conventional transvenous systems but this difference was not significant.^11^ Similarly, in a propensity score matched analysis of 193 patients with LLPMs and conventional devices, significant TR was more prevalent in transvenous than leadless devices (12% vs. 9%), but again, this difference was not significant.^24^

Given the limited evidence, it seems possible that LLPMs may better preserve tricuspid valve function after device implantation as unlike transvenous leads, they do not permanently cross the valve. However, LLPMs may—with unknown frequency—still adhere to the tricuspid valve and subvalvular apparatus, posing a challenge if device extraction is performed.^25^ Moreover, a functional TR component induced by the non‐physiologic RV stimulation may remain, despite the recently introduced more physiologic LLPMs (i.e., atrio‐ventricular synchronous LLPMs^26^).

Recent developments in conduction system pacing on the other hand, may also offer advantages over conventional RV apical pacing. His‐Bundle pacing permits ventricular stimulation without crossing the tricuspid valve in some patients. Thanks to the more physiologic ventricular activation, His‐bundle and left bundle branch pacing may reduce the functional component and decrease TR.^27^, ^28^

### 4.3 Limitations

This is an observational study with a limited sample size. TTE were gathered from different sources. TR during follow‐up may also have been influenced by other factors such as general disease progression, adaption of the drug regimen, inclusion of patients with pulmonary hypertension and alike. Thus, prevalence of significant TR before and after LLPM implantation should not be confused with the rate of new‐onset significant TR as some patients may show TR alterations during the follow‐up due to other reasons. Patients with AF were not excluded. The variable follow‐up duration and significant proportion of patients who underwent aortic valve replacement may also have skewed our results. Finally, our analysis is based on data after implantation of an LLPM of one specific manufacturer.

The meta‐analysis is limited by a relatively short available follow‐up period and includes studies with mostly retrospective observational design. Besides analyzing crude overall prevalence of significant TR, the incidence, mechanism and severity degree of new‐onset TR after LLPM implantation and the percentage of patients that showed TR worsening/improvement would also be of interest. However, only limited information is provided by the included studies regarding these issues. Moreover, different articles could not be included quantitatively in the analysis since they were not reporting data on TR severity in sufficient detail.^10^, ^23^

## 5 CONCLUSION

Changes in TR severity after LLPM implantation occur, but the majority of patients have unchanged or improved tricuspid valve function after implantation. A pooled analysis of available evidence does not indicate a significant increase in the prevalence of significant TR before and after LLPM implantation.

## Supporting information

Supplementary Figure 1: Funnel plot of the studies included in the meta analysis.
