# Supplementary figures and images for: Evolution of tricuspid valve regurgitation after implantation of a leadless pacemaker: A single center experience, systematic review, and meta‐analysis
Source: J Cardiovasc Electrophysiol. 2022 Jun 7;33(7):1617–27. doi: 10.1111/jce.15565 (PMC9545011; doi:10.1111/jce.15565)

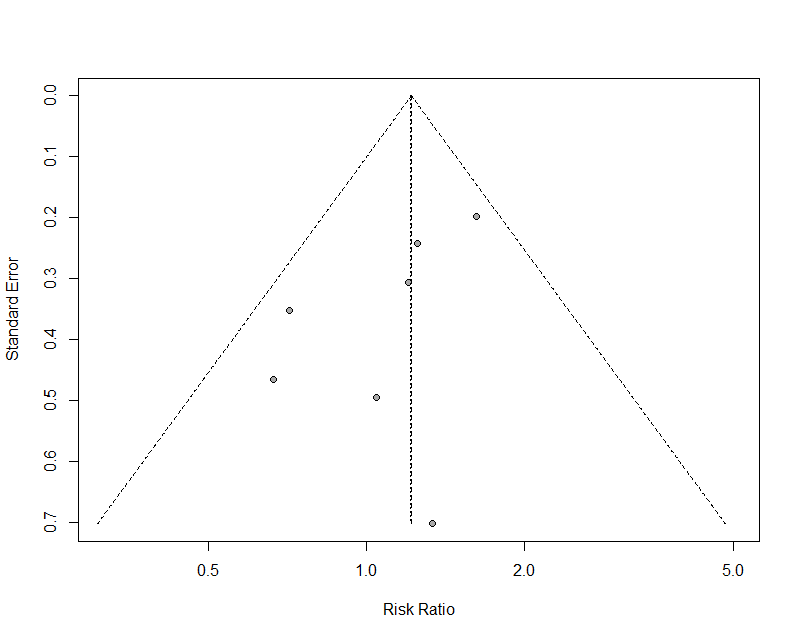

Supplement: Supplementary file 1 — Supplementary Figure 1: Funnel plot of the studies included in the meta analysis. [file JCE-33-1617-s001.tif]
